# Estimating the Prevalence and Characteristics of Patients Potentially Eligible for Lipoprotein(a)-Lowering Therapies in a Real-World Setting

**DOI:** 10.3390/biomedicines11123289

**Published:** 2023-12-12

**Authors:** Arrigo F. G. Cicero, Federica Fogacci, Marina Giovannini, Elisa Grandi, Sergio D’Addato, Claudio Borghi

**Affiliations:** 1 Hypertension and Cardiovascular Risk Factors Research Centre, Medical and Surgical Sciences Department, Alma Mater Studiorum University of Bologna, 40100 Bologna, Italy; arrigo.cicero@unibo.it (A.F.G.C.); marina.giovannini3@unibo.it (M.G.); elisa.grandi@unibo.it (E.G.); sergio.daddato@unibo.it (S.D.); claudio.borghi@unibo.it (C.B.); 2 Cardiovascular Medicine Unit, IRCCS Azienda Ospedaliero-Universitaria di Bologna, 40138 Bologna, Italy

**Keywords:** lipoprotein(a), cardiovascular diseases, ASCVD, lipid clinic, epidemiology

## Abstract

High lipoprotein(a) (Lp(a)) plasma levels are significantly associated with an increased risk of developing atherosclerotic cardiovascular diseases (ASCVD). The aim of this analysis was to estimate the prevalence and characteristics of patients potentially eligible for Lp(a)-lowering therapies in a real-world setting (i.e., patients with ASCVD and Lp(a) levels > 70 mg/dL). For this reason, we pooled data from a large cohort of Italian outpatients (N = 5961; men: 2879, women: 3982) with dyslipidemia. A binary logistic regression analysis was used to determine the significant predictors of ASCVD in the cohort, which were age (Odds Ratio (OR): 1.158, 95% Confidence Interval (CI): 1.114 to 1.203, *p* < 0.001), low-density lipoprotein cholesterol at entry (OR: 1.989, 95% CI: 1.080 to 1.198, *p* = 0.020) and Lp(a) (OR: 1.090, 95% CI: 1.074 to 1.107, *p* < 0.001). In our cohort, almost half of patients with ASCVD (44.7%) may be eligible to be treated with Lp(a)-lowering agents. Interestingly, patients who do not meet the treatment criteria despite high Lp(a) (50–70 mg/dL), respectively, account for 4.7% and 7.3% of those in primary and secondary ASCVD prevention. In conclusion, in our large cohort of outpatients with dyslipidemia, the prevalence of individuals with ASCVD and very high Lp(a) plasma levels is quite high, even with a conservative estimation.

## 1. Introduction

Lipoprotein(a) is a form of low-density lipoprotein (LDL) with an apolipoprotein(a) (apo(a)) with different length that is bound to the apolipoprotein B-100 (apo B-100). The length of apo(a) and the plasma levels of Lp(a) are strictly genetically determined [1]. In recent decades, several epidemiological studies have definitively shown that high Lp(a) levels are associated with a significant increase not only in the risk of atherosclerosis-related cardiovascular (ASCV) events (namely coronary artery disease (CAD), stroke and peripheral artery disease) [2] but also aortic stenosis [3] and atrial fibrillation [4].

A comprehensive meta-analysis of 75 cohort studies (*n.* 957,253) found that individuals in the top tertile of Lp(a) have a higher risk of all-cause mortality compared the bottom Lp(a) tertile, with a hazard ratio (HR) of 1.09 and a 95% Confidence Interval (CI) ranging from 1.01 to 1.18 in the general population and a HR of 1.18 and a 95% CI ranging from 1.04 to 1.34 in patients with ASCV disease (ASCVD) [5]. The HRs for ASCVD mortality were 1.33 (95%CI: 1.11 to 1.58) in the general population and 1.25 (95%CI: 1.10 to 1.43) in patients with ASCVD [5]. Interestingly, for each 50 mg/dL rise in Lp(a) plasma levels, a 31% and 15% greater risk of ASCV death was estimated, respectively, in the general population and in patients with ASCVD [5].

Currently, there is no effective way to manage patients with a high Lp(a) level [6]. The prothrombotic effect of Lp(a) can be partially counteracted via the use of antiplatelet drugs, but the evidence is greater in patients with ASCVD [7]. Emerging lipid-lowering drugs—namely small interfering ribonucleic acid (siRNA) agents olpasiran (LY3819469, SLN360, formerly known as AMG-890, ARO-LPA) and the second-generation antisense oligopeptide pelacarsen (also known as AKCEA-APO[a]-LRx)—are being developed to specifically interfere with Lp(a) synthesis by preventing the protein translation of apo(a) messenger RNA (mRNA) into apo(a) [8]. The ultimate objective of this therapy is to genetically silence LPA, reduce apo(a) production and lower the circulating Lp(a) levels, of consequence [9]. The available evidence shows that the monthly subcutaneous administration of these agents yields reductions in Lp(a) up to 95% that persist over time and are expected to be enough to optimize the risk of ASCVD, as far as reductions in Lp(a) plasma levels by 80–90% are expected to exert a clinically significant effect [10]. A relevant Lp(a)-lowering effect has been recently shown also in a phase I trial on an oral drug, muvalaplin, introducing a different option for targeting Lp(a) in clinical development [11]. Muvalaplin is the first oral agent specifically developed to disrupt Lp(a) formation by blocking the initial noncovalent interaction between apo(a) and apo B-100 [11]. Of course, it should be noted that once-daily administration of muvalaplin lowers Lp(a) levels to a lesser degree (~65%) than parenteral therapies currently in development, even though the molecule has not been associated with any serious adverse effects in the short term. Actually, all these new drugs targeting Lp(a) are very promising and also overall safe, as far as their cost-effectiveness will be definitively established as part of streamlining the health investments in ASCVD prevention [12]. Moreover, phase III pivotal CV outcome trials (CVOTs)—OCEAN(a) with olpasiran and Lp(a)HORIZON with pelacarsen—are ongoing to evaluate their efficacy in the secondary prevention of major ASCV events in patients with elevated Lp(a) [13].

In this context, the aim of our study was to estimate the prevalence and characteristics of patients potentially eligible for Lp(a)-lowering therapies in a real-world setting.

## 2. Methods

### 2.1. Study’s Design

For this study, we retrospectively reviewed and assessed 7349 clinical records of as many patients first evaluated at the lipid clinic of the University Hospital of Bologna (at entry and before optimizing lipid-lowering therapy) from January 2020 to August 2023. The clinical records included in this study belonged to patients not reaching the LDL-C goal (as defined by the 2019 European Society of Cardiology (ECS)/European Atherosclerosis Society (EAS) guidelines [14]) and/or with triglycerides (TG) greater than 150 mg/dL), for whom their Lp(a) plasma levels had been obtained at least once in life. We excluded clinical records with incomplete data, clinical records belonging to patients with dyslipidemia secondary to hypothyroidism and nephrotic syndrome and patients with dyslipidemia caused by treatment with steroids, anti-human immunodeficiency virus (HIV) drugs or atypical antipsychotics. Clinical records of patients younger than 40 years old were also excluded from the study.

Patients were defined as potentially eligible for treatment with Lp(a)-lowering drugs based on the contemporary presence of a Lp(a) value > 70 mg/dL and an ASCVD (CAD, ischemic stroke, peripheral obstructive arterial disease).

The study protocol was approved by the Institutional Ethical Board of the University Hospital of Bologna (code: LLD-RP2018) and performed in accordance with the ethical standards laid down in the 1964 Declaration of Helsinki and its later amendments. All involved patients voluntarily signed an informed consent form consenting to their inclusion in the study.

### 2.2. Assessments

#### 2.2.1. Clinical Data and Physical Assessments

The information included in the clinical records was age, smoking habits, anthropometric measurements, blood pressure values, laboratory data, family and personal history of ASCVD, presence of any systemic disease and the use of medication [15]. Following the classification of the American College of Cardiology (ACC) and the American Heart Association (AHA), the intensity of background statin therapy was reported as divided into 3 categories [16]. High-intensive statin use was defined as atorvastatin ≥ 40 mg or rosuvastatin ≥ 20 mg; moderate-intensity statin use was defined as atorvastatin ≤ 20 mg, rosuvastatin ≤ 10 mg, simvastatin ≥ 20 mg, pravastatin ≥ 40 mg, lovastatin ≥ 40 mg or fluvastatin 80 mg; low-intensity statin use was defined as simvastatin 10 mg, pravastatin ≤ 20 mg, lovastatin ≤ 20 mg or fluvastatin ≤ 40 mg.

The presence of ASCVD was self-reported and confirmed by patients’ clinical documentation, when available. Information about subclinical atherosclerosis for primary prevention was almost never available on the first visit. For this reason, it has almost never been taken into consideration in the risk stratification. Familial hypercholesterolemia (FH) was diagnosed using the Dutch Lipid Score (DLS) [17]. Polygenic hypercholesterolemia, familial combined hyperlipidaemia (FCH) and familial hypertriglyceridemia were diagnosed following the indication of the 2019 ESC/EAS guidelines [14].

Waist circumference (WC) was measured in a horizontal plane at the end of a normal expiration, at the midpoint between the inferior margin of the last rib and the superior iliac crest. Hip circumference was measured at the largest circumference around the buttocks. Height and weight were, respectively, measured to the nearest 0.1 cm and 0.1 kg, with subjects standing erect with their eyes directed straight while wearing light clothes and having bare feet. Body mass index (BMI) was calculated as body weight in kilograms, divided by height squared in meters (kg/m^2^).

Systolic (SBP) and diastolic (DBP) blood pressure were measured three times at a 1 min interval using a standard sphygmomanometer, with the subject in the seated position and after 5 min of quiet rest. The average value of the three measurements was taken as individual blood pressure value, as recommended by the international guidelines [18]. Pulse pressure (PP) was calculated as the difference between the SBP and DBP. The pulse pressure index (PPI) was calculated as the ratio between the PP and SBP. The mean arterial pressure was DBP + 1/3 (SBP − DBP). Hypertension diagnosis was made based on BP values higher than 140 and/or 90 mmHg or antihypertensive drug use.

#### 2.2.2. Laboratory Analysis and Derived Measures

Biochemical analyses were carried out on venous blood withdrawn after overnight fasting (at least 12 h). The serum was obtained via the addition of disodium ethylenediaminetetraacetate (Na_2_EDTA) (1 mg/mL) and blood centrifugation at 3000 RPM for 15 min at 25 °C. Immediately after centrifugation, trained personnel performed laboratory analyses according to the standardized methods. The following parameters were directly assessed: total cholesterol (TC), TG, high-density lipoprotein cholesterol (HDL-C), fasting plasma glucose (FPG), serum uric acid (SUA), creatinine, creatine phosphokinase (CPK), gamma-glutamyl transferase (gGT), alanine transaminase (ALT) and aspartate transaminase (AST).

The LDL-C was calculated using the Friedewald formula [19]. Very-low DL-C (VLDL-C) was obtained by dividing the TG by a factor of 5 [20]. When the plasma TG levels were higher than 400 mg/dL, the Sampson formula was used [21]. The atherogenic index was calculated as the ratio between non-HLD-C and HDL-C [22]. The glomerular filtration rate (eGFR) was estimated using the Chronic Kidney Disease Epidemiology Collaboration (CKD-epi) equation [23].

Plasma levels of Lp(a) were determined using an immunoturbidimetric assay [24].

The visceral adiposity index (VAI) was calculated using the following formulas: WC/[39.68 + (1.88 × BMI)] × (TG/1.03) × (1.31/HDL-C) for men and WC/[36.58 + (1.89 × BMI)] × (TG/0.81) × (1.52/HDL-C) for women, with both TG and HDL-C expressed as mMol/L [25]. The normality cut-off thresholds were 2.23 for patients aged between 40 and 42 years old, 1.92 for patients between 42 and 52 years old, 1.93 for patients aged 52–66 years old and 2.00 for patients > 66 years old [26].

### 2.3. Statistical Analysis

A full descriptive analysis was performed. The normal distribution of the continuous variables was tested using the Kolmogorov–Smirnov normality test. Normally distributed variables were compared using the Student’s *t*-test. When normal distribution was lacking, a Mann–Whitney-U test was applied. Categorical variables were compared using the Chi-square test—followed by the Fisher’s exact test. The analysis was repeated by sex and presence of ASCVD. Finally, a binary logistic regression analysis was performed to evaluate which predictors were most strongly associated with ASCVD in the study cohort. The parameters included in the logistic regression were age, sex, smoking habits (dichotomous), MAP, FPG, LDL-C, TG, Lp(a), gGT, SUA and eGFR. A *p*-value less than 0.05 was considered significant for all tests. All analyses were carried out using Statistical Package for Social Sciences (SPSS), version 25 for Windows.

## 3. Results

**Figure 1:**
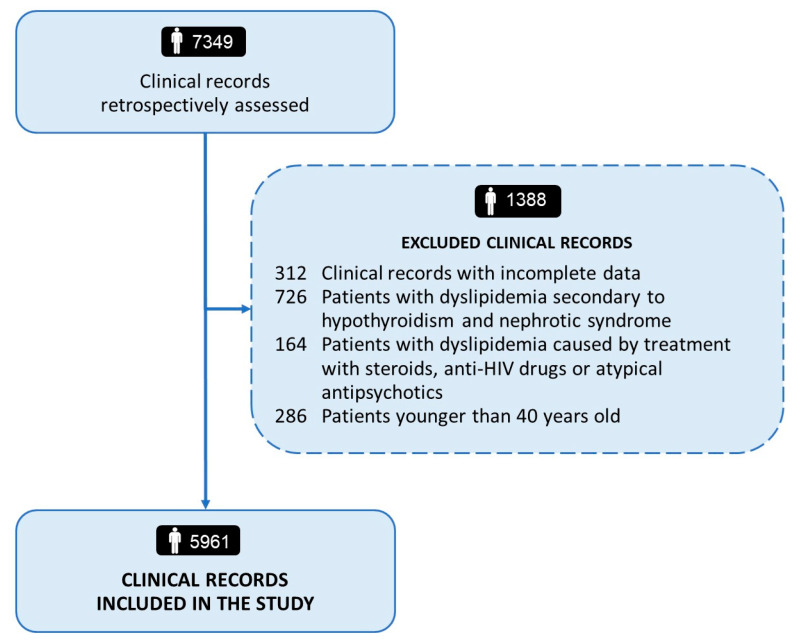
Flow diagram of study cohort.

After retrospectively reviewing and assessing the clinical records according to the pre-specified inclusion and exclusion criteria of this analysis, we identified and pooled data from 5961 outpatients (2879 men and 3982 women) referred to our lipid clinic. The study flowchart has been shown in Figure 1.

Current cigarette smoking was higher among men (*n.* 1166, corresponding to 40.5%) than women (*n*. 401, corresponding to 13%), as was former smoking (men: 600, 24.3%; women: 483, 19.8%).

The distribution of the main variables we investigated have been given in Table 1.

Briefly, the study cohort was characterized by a relatively high prevalence of overweight and hypertension. When the analysis was repeated by sex, the distribution of most variables was found to be significantly different between men and women (Table 1).

Descriptive statistics of variables by presence of ASCVD are reported in Table 2.

The Lp(a) plasma levels were significantly higher in patients with ASCVD (*p*-value < 0.05), except in patients affected by familial hypertriglyceridemia (*p*-value > 0.05) (Table 3).

Patients with normal Lp(a) values accounted for 79.5% of patients in primary prevention of ASCVD and for 76.8% of patients in secondary prevention of ASCVD. However, patients with high to very high levels of Lp(a) were more likely to be in secondary prevention of ASCVD (54.4% versus 8.8% of patients without any ASCVD; *p*-value < 0.001) (Table 4). Considering the main inclusion criteria of the ongoing phase III clinical trials (namely Lp(a)HORIZON and OCEAN(a)), the potential number of patients eligible for treatment with pelacarsen or olpasiran could account for 47.4% of all patients with ASCVD, in the real-world setting of a large lipid clinic (Table 4).

**Figure 2:**
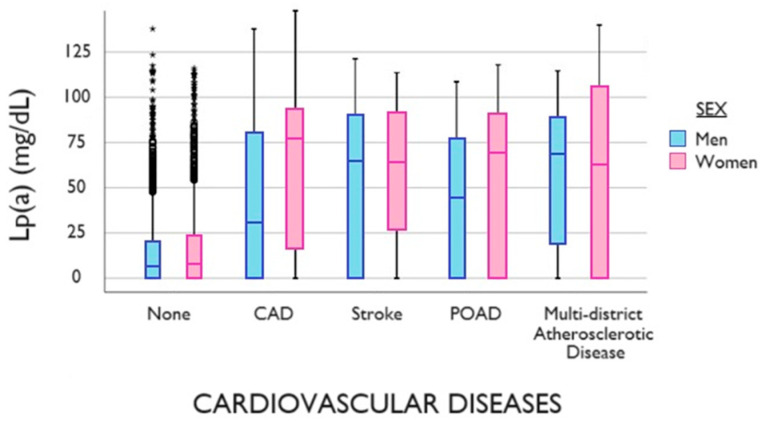
Distribution of Lp(a) in patients with or without ASCVD (box plots show median and interquartile interval by sex). CAD = coronary artery disease; Lp(a) = lipoprotein(a); POAD = peripheral obstructive artery disease.

The prevalence of ASCVD was 10.2% in men and 11.8% in women (*p*-value = 0.218). Among the patients attending the lipid clinic, only 312 men and 316 women reported having a CAD, 62 men and 80 women had already had a stroke and 42 men and 60 women suffered from peripheral obstructive artery disease and multidistrict events, without any statistically significant difference between sexes (*p*-value= 0.499) (Figure 2).

A binary logistic regression analysis found that significant predictors of ASCVD in our cohort of outpatients were age (Odds Ratio (OR): 1.158, 95% Confidence Interval (CI): 1.114 to 1.203, *p*-value < 0.001), LDL-C at entry (OR: 1.989, 95% CI: 1.080 to 1.198, *p*-value = 0.020) and Lp(a) (OR: 1.090, 95% CI: 1.074 to 1.107, *p*-value).

## 4. Discussion

Experts in CV prevention have great expectations for the newly developed selective Lp(a)-lowering treatment, whose effect, however, until now has mainly been tested in patients with very high Lp(a) plasma levels already complicated by ASCV events. Of course, this approach will magnify the cost–benefit ratio of a possible positive effect of these drugs on CV outcomes. On the other side, it is not yet clear how many patients will be eligible for that kind of treatment in different settings.

In our outpatient sample, the Lp(a) plasma levels are relatively high when compared to the values reported in the general population of the same geographic area [27,28], especially as regards patients with ASCVD. However, it must be acknowledged that our lipid clinic functions as secondary-level healthcare, which provides services to high-risk patients, and patients apparently resistant to first-level lipid-lowering treatments [29].

In our cohort, almost half of patients with ASCVD (44.7%) may be eligible to be treated with Lp(a)-lowering agents. Interestingly, patients who do not meet the treatment criteria despite high Lp(a) (50–70 nm/dL), respectively, account for 4.7% and the 7.3% of those in primary and secondary ASCVD prevention.

In recent decades, the epidemiological and pathophysiological link between Lp(a) plasma levels and the risk of ASCVD has been clearly defined [30,31,32]. However, Lp(a) dosage has not been routinely suggested in clinical practice for a long time, because of the lack of therapeutic options able to significantly decrease its plasma levels [33]. As clearly demonstrated by the EPIC-Norfolk data [31], a healthy lifestyle positively impacts the ASCVD risk of patients with high Lp(a), even if it does not affect the Lp(a) plasma levels [34]. Some nutraceuticals (including L-carnitine, curcumin and coenzyme Q10) mildly (≈−10%) improve this parameter, even though their effect is substantially irrelevant from both a clinical and prognostic point of view [35,36]. Statins do not lower Lp(a): they partly balance its negative effect on the ASCVD risk for concentrations < 50 mg/dL, but not for higher values [8,37]. Prolonged-release (PR) nicotinic acid is the only agent able to reduce Lp(a) by 20–30%, but it is not always well tolerated [38]. Moreover, its use has not been significantly associated with a reduced risk of ASCV events in the long term [39]. The antisense oligonucleotide Mipomersen directed at apo B-100 mRNA in the liver is also able to reduce Lp(a). However, its liver safety has been seriously questioned, so the drug is not approved in several countries [40]. Cholesteryl ester transfer protein (CETP) inhibitors increase the HDL fraction while decreasing the number of atherogenic non-HDL particles, such as Lp(a) [41]. According to the findings of a systematic review and meta-analysis of 10 randomized controlled clinical trials (including overall 34,781 patients), anacetrapib significantly lowers plasma Lp(a) by a weighted mean difference (WMD) of −13.35 (95%CI: −18.31 to −8.39) [42]. Recently, another CETP inhibitor, namely Evacetrapib, has also been shown to reduce Lp(a) by 30–40% over a 12-week period [43]. However, no one of these drugs has been associated with ASCVD risk reduction. In cohort studies, plasma proprotein convertase subtilisin/kexin type 9 (PCSK9) has been related to Lp(a) plasma levels [44]. On the other hand, PCSK9 inhibitors have been shown to mildly affect plasma levels of Lp(a) [45,46]. A recent network meta-analysis of 41 randomized controlled clinical studies with 17,601 participants concluded that the currently available PCSK9 inhibitors can significantly reduce Lp(a) (by up to 25.1%), with Alirocumab and Evolocumab being more effective than Inclisiran [47,48]. Remarkably, in both the FOURIER (Further Cardiovascular Outcomes Research with PCSK9 Inhibition in Subjects with Elevated Risk) trial and the ODYSSEY OUTCOMES (Evaluation of Cardiovascular Outcomes After an Acute Coronary Syndrome During Treatment With Alirocumab) trial, improvements in Lp(a) plasma levels have been associated with significant reductions in ASCV events regardless of LDL-C [49]. Unfortunately, this effect, although positive, rarely has a significant impact on the management of patients with Lp(a) plasma levels ranging from high to very high. For this reason, to date, in patients with progressive ASCVD and severe hyperlipoproteinemia(a), lipoprotein apheresis is still the most effective treatment, though it consists of an invasive, expensive, impractical and not widely available procedure [50]. Of course, even very low Lp(a) plasma levels could be associated with adverse health events in humans [51], but we do not yet know whether a marked plasma Lp(a) reduction would be safe in the long term.

Our study has a number of limitations. Our lipid clinic is highly specialized in patients with severe lipid disorders, while Lp(a) is yet rarely measured by general practitioners. For this reason, it is highly probable that patients undergoing Lp(a) tests have more severe clinical features. On the other hand, general practitioners also know that currently no effective treatment exists to reduce Lp(a), so they do not usually ask for specialist consultancy patients when they are able to reach patients’ LDL-C goals, as per the current guidelines [14]. This could partly explain the relatively small number of patients with Lp(a) plasma levels higher than 50 mg/dL in our cohort. Furthermore, the transversal design of the study does not allow us to conclude a cause–effect relationship between high Lp(a) levels and incidence of ASCVD, which, however, has already been clearly demonstrated in the literature [2]. The number of available parameters is relatively small and other confounding variables could have been considered in this analysis. Moreover, the prevalence of ASCVD in our cohort could have been underestimated because it has been often self-reported. Moreover, as we aimed to apply the inclusion criteria of the Lp(a)HORIZON and OCEAN(a) trials, we did not account for the presence of asymptomatic carotid disease and/or peripheral artery disease as CV complications [52,53]. This could partly explain the relatively low prevalence of patients affected by ASCVD in our cohort, compared to the ones reported by other lipid clinics [54]. Finally, the presence of autoimmune disorders—secondarily associated with hyperlipoproteinemia(a) —could be underestimated, since this was self-reported too.

## 5. Conclusions

In conclusion, in our large cohort of outpatients with dyslipidemia, the prevalence of individuals with ASCVD and very high Lp(a) plasma levels is quite high, even with a conservative estimation. The present observations could have relevant pharmacoeconomic implications when new Lp(a)-lowering drugs become available. 

## Figures and Tables

**Table 1:** Distribution of the investigated variables by sex. Data have been reported as mean ± standard deviation (SD), besides age, which has been reported as mean and variation range, and TG and Lp(a), which have been reported as median and interquartile range. Normally distributed variables have been compared using a *t*-test for independent samples; non-normally distributed variables have been compared using the Mann–Whitney-U test.

Parameters	Entire Cohort (*n*. 5961)	Men (*n.* 2879)	Women (*n.* 3982)	*p*-Value
Age (years)	60.3 (40–87)	60.5 (40–84)	59.4 (41–89)	0.123
BMI (kg/m^2^)	26.4 ± 4.3	26.5 ± 3.7	26.3 ± 4.7	0.053
Waist Circumference (cm)	93.6 ± 12.9	98.3 ± 10.8	89.2 ± 13.1	<0.001
Waist/Hip Ratio	0.93 ± 0.08	0.96 ± 0.08	0.88 ± 0.08	0.011
VAI	4.6 ± 4.0	4.3 ± 4.0	4.7 ± 4.2	0.044
SBP (mmHg)	141.4 ± 11.5	139.7 ± 10.2	142.9 ± 12.5	<0.011
DBP (mmHg)	82.6 ± 6.4	82.6 ± 6.0	82.2 ± 6.8	0.020
PP (mmHg)	58.6 ± 8.1	56.6 ± 8.9	60.5 ± 9.8	<0.001
MAP (mmHg)	103.4 ± 7.1	102.9 ± 6.5	103.8 ± 7.5	0.009
PP Index	0.7 ± 1.2	0.7 ± 1.6	0.7 ± 0.9	0.460
TC (mg/dL)	231.1 ± 43.1	225.1 ± 41.8	236.7 ± 43.5	<0.001
TG (mg/dL)	125.4 (42.4–214.7)	134.5 (48.2–254.9)	113.1 (42.7–183.9)	<0.001
HDL-C (mg/dL)	54.8 ± 7.2	51.1 ± 6.5	58.1 ± 7.0	<0.001
Non-HDL-C (mg/dL)	176.4 ± 43.3	173.9 ± 41.9	178.6 ± 44.4	<0.001
LDL-C (mg/dL)	152.0 ± 39.7	147.8 ± 38.3	155.8 ± 40.4	<0.001
VLDL-C (mg/dL)	24.7 ± 8.2	26.6 ± 9.5	22.9 ± 6.3	<0.001
Lipoprotein(a) (mg/dL)	25.4 (3.1–55.5)	24.3 (2.9–49.8)	27.1 (3.3–59.2)	<0.001
Atherogenic Index	2.9 ± 1.6	3.1 ± 1.7	2.7 ± 1.4	<0.001
FPG (mg/dL)	90.2 ± 21.1	92.1 ± 21.7	88.3 ± 20.9	<0.001
SUA (mg/dL)	4.9 ± 1.6	5.5 ± 1.4	4.3 ± 1.4	<0.001
gGT (mg/dL)	28.1 ± 18.8	34.5 ± 21.8	22.1 ± 14.2	<0.001
eGFR (mL/min)	73.5 ± 11.7	71.8 ± 13.5	75.7 ± 11.8	<0.001

BMI = Body Mass Index, eGFR = Estimated Glomerular Filtration Rate, FPG = Fasting Plasma Glucose, DBP = Diastolic Blood Pressure, gGT = Gamma-Glutamyl-Transferase, HDL-C = High-Density Lipoprotein Cholesterol, LDL-C = Low-Density Lipoprotein Cholesterol, MAP = Mean Arterial Pressure, *n.* = Number of Individuals, PP = Pulse Pressure, SBP = Systolic Blood Pressure, SUA = Serum Uric Acid, TC = Total Cholesterol, TG = Triglycerides, VAI = Visceral Adiposity Index, VLDL-C = Very Low-Density Lipoprotein Cholesterol.

**Table 2:** Distribution of the investigated variables by presence of ASCVD. Data have been reported as mean ± standard deviation (SD), besides age, which has been reported as mean and variation range, and TG and Lp(a), which have been reported as median and interquartile range. Normally distributed variables have been compared using a *t*-test for independent samples; non-normally distributed variables have been compared using the Mann–Whitney-U test.

Parameters	Primary Prevention of ASCVD (*n*. 5089)	Secondary Prevention of ASCVD (*n.* 872)	*p*-Value
Age (years)	60.3 (40–87)	70.1 (49–86)	0.009
Background lipid-lowering therapy			
Statin (*n.*; %)	3276 (64.4)	801 (91.6)	<0.001
High-intensity dosage (*n.*; %)	437 (13.3)	253 (31.6)	<0.001
Moderate-intensity dosage (*n.*; %)	2199 (67.2)	466 (58.2)	<0.001
Low-intensity dosage (*n.*; %)	640 (18.5)	82 (10.2)	<0.001
Ezetimibe (*n.*; %)	720 (14.1)	482 (55.3)	<0.001
BMI (kg/m^2^)	26.4 ± 4.3	26.5 ± 4.5	0.270
Waist circumference (cm)	93.5 ± 12.9	94.9 ± 12.5	0.517
Waist/hip ratio	0.92 ± 0.09	0.91 ± 0.08	0.597
VAI	4.5 ± 4.1	4.7 ± 4.3	0.668
SBP (mmHg)	140.8 ± 11.2	150.1 ± 11.3	<0.001
DBP (mmHg)	82.6 ± 6.1	82.1 ± 6.4	0.473
PP (mmHg)	58.1 ± 8.7	67.9 ± 10.3	<0.001
MAP (mmHg)	103.2 ± 6.6	106.4 ± 7.1	<0.001
PP index	0.7 ± 1.6	0.7 ± 0.8	0.344
TC (mg/dL)	230.8 ± 42.8	236.5 ± 46.6	0.031
TG (mg/dL)	121.4 (49.3–232.8)	134.2 (55.8–224.8)	0.074
HDL-C (mg/dL)	54.7 ± 7.1	55.7 ± 7.0	0.312
Non-HDL-C (mg/dL)	176.1 ± 43.1	180.8 ± 46.3	0.243
LDL-C (mg/dL)	151.8 ± 39.4	155.9 ± 43.4	0.125
VLDL-C (mg/dL)	26.2 ± 8.5	26.1 ± 7.8	0.070
Lipoprotein(a) (mg/dL)	21.4 (3.4–52.8)	73.8 (24.5–131.8)	<0.001
Atherogenic index	3.0 ± 1.6	2.8 ± 1.5	0.093
FPG (mg/dL)	89.9 ± 21.1	93.4 ± 25.7	0.003
SUA (mg/dL)	4.9 ± 1.6	5.2 ± 1.4	<0.001
gGT (mg/dL)	27.6 ± 16.2	34.4 ± 15.3	<0.001
eGFR (mL/min)	77.1 ± 12.6	76.8 ± 11.4	0.294

ASCVD = Atherosclerotic Cardiovascular Disease, BMI = Body Mass Index, eGFR = Estimated Glomerular Filtration Rate, FPG = Fasting Plasma Glucose, DBP = Diastolic Blood Pressure, gGT = Gamma-Glutamyl-Transferase, HDL-C = High-Density Lipoprotein Cholesterol, LDL-C = Low-Density Lipoprotein Cholesterol, MAP = Mean Arterial Pressure, *n.* = Number of Individuals, PP = Pulse Pressure, SBP = Systolic Blood Pressure, SUA = Serum Uric Acid, TC = Total Cholesterol, TG = Triglycerides, VAI = Visceral Adiposity Index, VLDL-C = Very Low-Density Lipoprotein Cholesterol.

**Table 3:** Lp(a) plasma levels by presence of ASCVD. Data have been reported as media and interquartile range; subgroups have been compared using Mann–Whitney U test.

	Lp(a) (mg/dL)	
	Primary Prevention of ASCVD	Secondary Prevention of ASCVD
Polygenic hypercholesterolemia (*n.* 1749)	21.3 (3.8–41.9)	69.8 (41.4–122.4) *
HeFH (*n.* 339)	31.1 (13.4–44.1)	88.2 (51.9–111.8) *
FCH (*n.* 1929)	29.2 (4.2–61.1)	90.3 (44.2–123.8) *
Diabetic dyslipidaemia (*n.* 1022)	33.4 (4.9–61.9)	87.8 (49.1–112.9) *
Metabolic syndrome (*n.* 580)	10.7 (4.2–18.4)	44.9 (29.4–63.4) *
Familial hypertriglyceridemia (*n.* 238)	13.3 (3.4–26.4)	17.3 (8.4–25.5)
Others (*n.* 104)	18.4 (3.3–32.2)	46.9 (12.8–63.9) *

* *p*-value < 0.05 versus primary prevention. ASCVD = Atherosclerotic Cardiovascular Disease, FCH = Familial Combined Hyperlipidaemia, HeFH = Heterozygous Familial Hypercholesterolemia, *n.* = Number of Individuals.

**Table 4:** Distribution of Lp(a) subgroups by primary and secondary prevention patients.

		Lp(a) Subgroups				Total
		<30 mg/dL	30–49 mg/dL	50–70 mg/dL	>70 mg/dL	
Primary prevention of ASCVD	*n.*	4092	614	221	162	5089
	%	80.4%	12.1%	4.3%	3.1%	100%
Secondary prevention of ASCVD	*n.*	384	12	64	412	872
	%	44.1%*	1.2%*	7.3%*	47.4% *	100%
Total	*n.*	4476	626	285	572	5961
	%	76.8%	11.9%	4.9%	6.4%	100%

* *p*-value < 0.05 versus primary prevention of ASCVD (Chi-square test). ASCVD = Atherosclerotic Cardiovascular Disease, Lp(a) = Lipoprotein(a), *n.* = Number of Individuals.

## Data Availability

Data supporting the findings of this analysis are available from the University of Bologna. Data are available from the authors with the permission of the University of Bologna.
